# Dissemination of clinical and scientific practice through social media: a SIAARTI consensus-based document

**DOI:** 10.1186/s44158-024-00157-3

**Published:** 2024-03-19

**Authors:** Andrea Cortegiani, Denise Battaglini, Giovanna Amato, Astrid Ursula Behr, Katia Donadello, Sharon Einav, Maria Grazia Frigo, Giorgio Fullin, Alberto Giannini, Mariachiara Ippolito, Franco Marozzi, Roberta Monzani, Gianpaola Monti, Marcus J. Schultz, Vito Torrano, Gianluca Villa, Antonino Giarratano

**Affiliations:** 1 Department of Precision Medicine in Medical, Surgical and Critical Care (Me.Pre.C.C.), University of Palermo, Palermo, Italy; 2 Department of Anesthesia, Analgesia, Intensive Care and Emergency, University Hospital Policlinico Paolo Giaccone, Palermo, Italy; 3 Anaesthesia and Intensive Care, IRCCS for Oncology and Neuroscience, San Martino Policlinico Hospital, Genoa, Italy; 4 Arnas Civico Di Cristina Benfratelli UOC Centro Regionale Trapianti Resso UOC Anestesia, Rianimazione e, Terapia Intensiva AOU Policlinico Giaccone, Palermo, Italy; 5 Department of Anaesthesiology and Intensive Care, Camposampiero Hospital, ULSS 6 Euganea, Padua, Italy; 6 Department of Surgery, Dentistry, Gynaecology and Paediatrics, University of Verona, Verona, 37129 Italy; 7 Anesthesia and Intensive Care B Unit, University Hospital Integrated Trust of Verona, Verona, 37134 Italy; 8 Faculty of Medicine, Hebrew University, Jerusalem, Israel; 9 Maccabi Healthcare Services, Hod HaSharon, Israel; 10 San Giovanni Calibita Fatebenefratelli Hospital, Rome, Italy; 11 Department of Anesthesia Intensive Care, Santa Maria dei battuti Ca’ Foncello Hospital, Treviso, Italy; 12 Unit of Pediatric Anesthesia and Intensive Care, Children’s Hospital ASST Spedali Civili Di Brescia, Brescia, Italy; 13 Italian Society of Legal and Insurance Medicine (SIMLA), Rome, Italy; 14 Department of Anaesthesiology and Intensive Care, IRCCS Humanitas Research Hospital, Milan, 20089 Italy; 15 Anestesia E Rianimazione Postchirurgica E Dei Trapianti Addominali, ASST Grande Ospedale Metropolitano Niguarda, Milan, Italy; 16 Department of Intensive Care, Amsterdam University Medical Centers Location AMC, Meibergdreef 9, Amsterdam, 1105 AZ The Netherlands; 17 Department of Emergency and Urgency, Anesthesia and Intensive Care 1, ASST Grande Ospedale Metropolitano Niguarda, Milan, Italy; 18 Department of Health Sciences Section of Anesthesia, Intensive Care and Pain Medicine, University of Florence, Florence, Italy; 19 Department of Anesthesia and Intensive Care Section of Oncological Anesthesia and Intensive Care, Azienda Ospedaliero Universitaria Careggi, Florence, Italy

**Keywords:** Communication, social-media, scientific dissemination, anaesthesia, intensive care, consensus document

## Abstract

**Background:**

Dissemination of medical practice and scientific information through social media (SoMe) by clinicians and researchers is increasing. Broad exposure of information can promote connectivity within the scientific community, overcome barriers to access to sources, increase debate, and reveal layperson perspectives and preferences. On the other hand, practices lacking scientific evidence may also be promoted, laypeople may misunderstand the professional message, and clinician may suffer erosion of professional status. The aim of this project was to enhance awareness and advise the anesthesia community and clinicians at large about the potential risks advocate for responsible use of SoMe to disseminate information related to medical practices and knowledge.

**Methods:**

A modified Delphi process with prespecified consensus criteria was conducted among a multidisciplinary panel of experts, including anesthesiologists-intensivists, clinical psychologists, and forensic medicine specialists. Six items were identified: Ethics and deontological principles, the practice of sharing information via social media, legal aspects, psychological aspects, self-promotion, and criteria for appropriate dissemination. Statements and rationales were produced and subjected to blinded panelists’ votes. After reaching consensus, a document was written which then underwent external review by experts uninvolved in the consensus process. The project was promoted by the Italian Society of Anesthesia Analgesia Resuscitation and Intensive Care (SIAARTI).

**Results:**

Twelve statements were produced, and consensus was achieved for all. The panel concluded that the general principles guiding dissemination of professional information via SoMe must remain in line with the general principles of ethics, deontology, and scientific validity that guide the medical profession and science in general. Professional equity must be maintained while communicating via SoMe. Medical practices lacking support by scientific evidence should not be disseminated. Patients’ informed consent must be obtained before dissemination of information, images, or data. Self-promotion must not be prioritized over any of these principles.

**Conclusions:**

When sharing medical practices and scientific information on SoMe, healthcare professionals are advised to act conscientiously and ethically. Local regulations should be adhered to. Institutional training on the potential risks and proper of SoMe for such purpose may contribute to preservation of professional integrity.

## Introduction

Scientific dissemination, defined as communication of knowledge, research findings, and discoveries to a range of audiences in a clear, accessible, and meaningful way, has a long history. Its origin is conventionally dated to 6 March 1665, with the publication of the text *Philosophical Transactions of the Royal Society*. Since then, scientific dissemination has been carried out through official and specialized channels and means, such as scientific societies and academic publishing, and through texts and journals or oral presentations at congresses [1]. Over time, the scientific community developed a set of formal and informal rules and a code of ethics that researchers, scientists, and practitioners in scientific fields (including healthcare professionals) must adhere to when disseminating scientific information. Dissemination of practice and research required proof of scientific method, reproducibility, and guarantees for the quality and ethics of the process and end product (https://publicationethics.org/). A *gentlemen’s agreement* of sorts was established between the producers of the data and information to be disseminated, the scientific publishers, and the reference societies. The latter enact and apply common rules and a code of ethics through the peer review process and the judgement of *super partes* experts (e.g., scientific editors and evaluation commissions) to ensure as best possible that the recipient of the information, i.e., the reader, receives a product that enhances their professionalism. Proper scientific dissemination is particularly important in the biomedical sciences, where the end product must not only increase professionalism but also align with ethical principles, animal rights, and the Hippocratic oath. In critical care and anesthesiology, much of the disseminated information pertains to critically ill or unconscious patients, which makes these requirements even more important.

Social media (SoMe) has substantially increased scientific dissemination to a wider audience and has introduced new forms of information presentation (e.g., infographics, blogs) [2, 3]. Scientific journals and societies established a convention for disseminating research results and scientific material, produced in accordance with methodological rigor and ethical codes, through SoMe (*post-production* dissemination). Indeed, healthcare professional has started to properly use SoMe by disseminating scientific information, facilitating the contact between medical community and scientific sources. In many cases, dissemination is accompanied by proper description, interpretation, or even simplification of scientific data as a form of divulgation. The increased exposure of the general public to scientific sources through SoMe has undeniably facilitated rapid scientific updates, the application of knowledge, and the cultural and professional enrichment of a larger audience (both medical and lay) than traditional means of communication [4, 5]. This should also be seen as a milestone in evidence dissemination, since it helps overcoming barriers that may preclude access to scientific sources (i.e., subscription-based access to journal articles, clinical burden that may reduce time for scientific update). On the other hand, SoMe may favor the spread of uncontrolled medical practices and information, bypassing the *gentlemen*’*s agreement* [6]. This occurs through direct contact between healthcare professionals producing “information” and members of the public [7]. The escalation of this phenomenon has been accompanied by “revelations” of unsafe practices lacking scientific support and failing to meet national and international safety standards. Social dynamics have fueled the widespread dissemination (*virality*) of some of these practices, capturing the public interest before the usual safeguards of scientific dissemination are employed. The victims of such dissemination are often the patients subjected to unproven practices. Their need for care places them at a disadvantage when the professional discloses or misrepresents information related to ethics, safety, dignity, and outcomes. This holds true regardless of the perceived success of the practice and the presumed “satisfaction” expressed for its “success.” In these circumstances, efficacy, safety, and patient satisfaction are misleading terms. These outcomes must be upheld by the results of studies conducted at the accepted level of scientific rigor. Only through rigorous adherence to scientific method can a medical practice be suitably evaluated and its validity proven. Healthcare professionals may even propagate such information becoming themselves victims of the *virality* and effectiveness of the media content. In consideration of the abovementioned issues, clinicians must be aware of the current advantages and the potential challenges related to the diffusion of scientific information through SoMe.

This consensus document aims to (i) enhance awareness and advise the anesthesia community and clinicians at large about the potential risks linked to the use of SoMe and (ii) advocate for responsible and professionally appropriate use of SoMe to disseminate information related to medical practices and scientific knowledge.

## Methodology

The methodology employed for achieving the consensus described in this document aligns with the current regulations of the Italian Society of Anesthesia, Analgesia, Resuscitation and Intensive Care (Società Italiana di Anestesia Analgesia Rianimazione e Terapia Intensiva — SIAARTI) for consensus-based good clinical practice documents. Specifically, the process was comprised of the following steps:

- Selection of the topic by the SIAARTI board of directors, following internal discussions and input from the affiliates
- Outlining of the planned methodology by an anesthesiologist-intensivist with methodology expertise (AC). This methodologist also coordinated the consensus process.
- Selection of a multidisciplinary panel of experts, including anesthesiologists-intensivists, clinical psychologists, and forensic medicine specialists basing on indication by scientific societies involved, roles and expertise related to the topic of the document, balancing age, career status, and gender representation
- Creation of a list of items by the coordinator after a preliminary informal discussion with the panelists about optional topics and preferences
- Assignment of item priority by panelist votes (deidentified and blinded to other votes), including free text comments for suggesting subsequent revision — this process was coordinated, and the comment was pooled by the methodologist.
- Assignment of panelists to one or more items based on expertise, skills, role, and career placement — by the methodologist
- Formulation of statements with their rationales by the groups of panelists through round table discussion
- Blind voting rounds
- Drafting of a manuscript for internal review — conducted by all panelists
- External critical review for content and method validity, performed by physician not involved in the panelists group
- Scientific dissemination and publication

Selection of the multidisciplinary panel was performed with the Bioethics Section and Communication Committee of SIAARTI, the Italian Society of Legal Medicine and Insurance (Società Italiana di Medicina Legale e delle Assicurazioni — SIMLA), and SIAARTI board members. A systematic review of the literature and assessment of the quality of the evidence were not planned. We acknowledge the lack of patient or public representative, nurses, or midwives in the panel as a limitation of this project.

Panel members sought and assessed literature to substantiate or negate their statements and rationales, and if the balance was considered sufficient by the majority to substantiate the statement, this evidence was incorporated as references within the text.

Although two rounds of online voting were allowed for, the second voting round was never necessary since all 12 statements reached consensus in the first round. All panel members were blinded to other panelist votes. Opinions were expressed using Likert scales in accordance with the RAND/UCLA method (lowest score, 1 = strongly disagree, highest score, 9 = strongly agree). This scale was then divided into tertiles: 1–3 implied rejection/disagreement (“not appropriate”), 4–6 implied “uncertainty,” and 7–9 implied agreement/support (“appropriate”). Consensus was reached when at least 75% of respondents agreed on a score within the same tertile, and the median score was within the same tertile. The consensus tertile was determined by the position of the median. Twelve statements were produced and voted. One statement (item 5) was afterwards reported in a narrative form, after external revision, maintaining the same wording and overall concepts, with the approval of the whole panel.

## Main text

### Ethics and deontological principles

#### Statements

1.1 The principles of *beneficence* and *non-maleficence* guide health professionals in all aspects of their professional activities, including the dissemination of health information. These principles carry the expectation that health professionals promote only therapies or treatments that have successfully undergone clinical trials and validation and have received endorsement from the scientific community.

$$\left[\mathrm{Median}\;\mathrm{score}:\;9\;(IQR\;7-9)\;\mathrm{agreement}:\;100\%\right]$$

1.2 Healthcare professionals should employ and discuss only clinically appropriate treatments, describing them in all circumstances with prudence, truthfulness, clarity, and honesty. It is essential to avoid conveying ambiguous or misleading messages.

$$\left[\mathrm{Median}\;\mathrm{score}:\;9\;(IQR\;7-9);\;\mathrm{agreement}:\;100\%\right]$$

1.3 Health treatments disseminated through SoMe should consistently be accompanied by references to scientific sources, primarily in peer-reviewed journals. The dissemination process must be accompanied by respect for patient decision-making autonomy.

$$\left[\mathrm{Median}\;\mathrm{score}:\;9\;(IQR\;7-9);\;\mathrm{agreement}:\;100\%\right]$$

#### Rationale

In all circumstances, medical personnel operate not only in accordance with the law but also with ethical and deontological principles [8]. The ethical principles that guide the dissemination of medical practice through SoMe are not different from the more general principles that guide other aspects of the profession. These are grouped into four core principles of equal importance: beneficence (to act for the benefit of the patient), non-maleficence (not to inflict harm intentionally), justice (appropriate allocation of resources), and autonomy (self-determination of both the health professional and the patient). Full compliance with these principles requires up-to-date knowledge of practices validated by the scientific community [9]. Codes of ethics fully embrace this approach, and this approach is articulated in various aspects of health professionals’ professional lives, including scientific dissemination. For example, Article 35 of the World Medical Association’s Code of Ethics emphasizes the significance of medical contributions to health literacy and education, advising caution when discussing new discoveries, technologies, or treatments in nonprofessional public settings [10]. Several articles within the Italian Code of Medical Ethics [11] address matters concerning scientific dissemination:

- Requiring that healthcare professionals keep up to date
- Mandating that clinicians disseminate only practices that have been scientifically and clinically documented
- Ensuring that patients are not deprived of scientifically proven treatments
- Emphasizing the central importance of informed consent
- Restricting advertising only to established practices and prohibiting the creation of unfounded expectations and illusory hopes

### Scientific evidence, clinical practice, and sharing of information through social media

#### Statements

2.1. Effective evidence-based scientific dissemination through SoMe requires simultaneous sharing of the scientific sources cited, thereby ensuring the appropriateness of a given clinical practice. Dissemination must be guided by both general ethics and medical professional ethics. Public sharing of images and techniques requires ethics committee approval as such approval ensures the respect of the rights and privacy of the individuals involved.

- $$\left[\mathrm{Median}\;\mathrm{score}:\;9\;(IQR\;7-9);\;\mathrm{agreement}:\;80\%\right]$$

#### Rationale

As part of continuing professional development, practitioners need access to relevant, high-quality research. Evidence-based practice refers to individual practices or treatments that are considered effective and safe based on scientific evidence. An “evidence-based” treatment or practice must be supported by data published in peer-review journals. Information provided by an original clinical research study, review, or evidence-based guidelines should be considered scientific information. In particular as follows:

- Evidence-based medicine (EBM) is *the conscientious, explicit, and judicious use of the best current evidence in making decisions about individual patient care* [12].
- Evidence-based (or research-based) practices are developed based on the best available research in the field. This means that users can be confident that there is a robust scientific basis for the use of the strategies and activities described. Alternatives, including innovative techniques, must be considered as *unproven clinical practice* (e.g., images, techniques) unless supported by appropriate scientific literature.

Appropriate evidence-based scientific dissemination through SoMe should be accompanied by disclosure and dissemination of the scientific sources used to demonstrate the appropriateness of the particular clinical practice at the same time [13]. Healthcare professionals must examine their scientific hypothesis according to a rigorous clinical trial protocol, following current regulations and ethics boards approval. Sharing a hypothesis on SoMe platforms must not be considered a legitimate means of bypassing proof of effectiveness and safety of any medical practice, technique, tool, or procedure.

Ethical regulations must also govern the dissemination of images and techniques, including the need to receive approval by an ethics committee that ensures the rights and privacy of those involved. In particular, the panel considers it mandatory to obtain patient’s informed consent prior to the distribution of any material/s that may or may not lead to their identification.

Clinical practices that are not evidence based (e.g., off-label use of drugs in terms of type, dosages preparation and route of administration, practices or procedures not supported by scientific evidence or *good medical practice*) must not be promoted. When such methods and practices are employed, the patients, often frail or suffering from comorbidities, are treated according to the principle of exceptionality rather than evidence-based medicine. Creating international and national guidelines, setting minimum standards for disseminating science via nontraditional channels, providing training on scientific communication, and enacting legislation can raise awareness about the risks of sharing unproven information [14].

### Legal aspects of sharing clinical content through social media

#### Statements

3.1. Communication through SoMe must strictly adhere to current national legislation. The dissemination of diagnostic-therapeutic interventions on the web must be avoided when conducted contrary to established rules, particularly if such action could potentially injure patients or violate their rights to information and privacy.

- $$\left[\mathrm{Median}\;\mathrm{score}:\;9\;(IQR\;7-9);\;\mathrm{agreement}:\;80\%\right]$$

#### Rationale

There is a proliferation of information, via social networks and the Internet, on questionable diagnostic and therapeutic interventions that are not supported by adequate scientific evidence, *good medical practice* and bioethical principles. Such practices may constitute a breach of legal principles, potentially qualifying as an offense against the person, subject to prosecution, with potential repercussions in terms of compensation to be adjudicated within a civil context. Given that SoMe transcends borders, whereas legislation is bound by them, this can be challenging. While local regulations should be applied according to place of residence, discrepancies may arise. For instance, one country might allow a degree of flexibility on dissemination practices, whereas another might offer none at all. Similarly, different standards may apply to informed consent regarding online distribution of images of patients who might or may not be identifiable.

### Psychological aspects

#### Statements

4.1 Nonscientific dissemination of content on web platforms is used by health professionals to share their knowledge and make information accessible to individuals outside the medical community. The characteristics of such communications (i.e., the short, fragmented, or disorganized text that is typical of the dialogic and multimedia nature of SoMe) may create ambiguity. Ambiguous or insufficient information can disintegrate the already fragile trust between non-healthcare professionals/patients and healthcare providers.

$$\left[\mathrm{Median}\;\mathrm{score}:\;9\;(IQR\;7-9);\;\mathrm{agreement}:\;91.6\%\right]$$

4.2 Attention to detail is important when sharing research or the concepts underlying medical practice on channels accessible to the general public, as interpretation largely depends on the health literacy of the reader.

$$\left[\mathrm{Median}\;\mathrm{score}:\;9\;(IQR\;7-9);\;\mathrm{agreement}:\;100\%\right]$$

4.3 Communication strategies should prioritize the *reliability* and c*redibility* of evidence-based content. This approach may facilitate the dissemination of scientific knowledge through SoMe while maintaining the professionalism and authority of the clinician and/or the institution sharing the information. Within this framework, shared content should be supported by appropriate specific references.

$$\left[\mathrm{Median}\;\mathrm{score}:\;9\;(IQR\;7-9);\;\mathrm{agreement}:\;91.5\%\right]$$

#### Rationale

Sharing content on SoMe has significantly redefined cultural, professional, and organizational interactions within the health sector, leading to evident changes in conventional methods of health and illness management and the communication dynamics within the doctor-patient relationship. Information now has a collaborative character. Medical science is not exempt from this process of *democratization of knowledge*, which promotes the empowerment and involvement of the non-professional/patient and, on the other hand, enables continuous training and updating of professionals [15].

### Dissemination

Through the dissemination of scientific content on web platforms, doctors simplify their knowledge, making it accessible to a broader audience. However, oversimplification and dramatization (i.e., transforming science or clinical practice into a show), which capture public attention, may come at the expense of losing the nuances of the scientific data and information or of passing on an implicitly wrong message. The language used in SoMe for medical content is often concise, sometimes fragmented, and may be disorganized. This may create message ambiguity or even mislead, both of which could lead to legal action.

Patients are nowadays akin to consumers, and they assert their right to know, evaluate, and maintain a sense of control over their illness. Gleaning knowledge from web sources may bridge the information asymmetry between doctor and patient and even tilt the power dynamic in favor of the non-professional/patient. This may lead to a transition from evidence-based medicine to narrative-based medicine, shaped by the *subjectivation* of knowledge acquired on the web, which in many cases is also insufficiently filtered because it is not mediated by specialists.

### Psychosocial impact

The scientific community unanimously recognizes the value of SoMe in doctor-patient communication. Such use can increase education and awareness and create a culture of prevention in the field of health. In this sense, the Web is becoming a relational network, a space for exchange and shared education, essentially humanizing medical science.

However, large amounts of information, even if biased, inaccurate, or tainted by conflicts of interest, remain on the Web indefinitely, in the frequent dichotomy between media rumors and institutional flows. Such information may activate individual mechanisms of simplification of reality, heuristics that can interfere with personal decision-making processes and cause confirmation bias (i.e., the choice of information that aligns best with personal preferences) or *cyberchondria* (i.e., the need to “know more” about symptoms).

In addition, repetitive instances of *amateurism* can undermine the reliability and truthfulness of shared content by calling into question the credibility of medicine and science and encouraging the active participation of non-specialists in the healthcare process. When the need for clear information and reliable sources is greatest, as is often the case when the stakes are high and the information is ambiguous and insufficient, “fake news” proliferates, undermining the already low existing level of trust in healthcare professionals and institutions [16–18].

### Responsibility

Improper use of web platforms or social networks exposes practitioners to the risk of compromising the traditional doctor-patient relationship. In extreme cases, SoMe may be sued to shame the clinician. Patient access to the practitioner’s personal content blurs the boundaries of the clinical relationship. This may lead to creation of informal situations wherein mechanisms of mistrust in the medical profession may be triggered. Public exposure of the personal details and opinions of the clinician may significantly influence patient satisfaction, potentially creating either dismissal or a “false” sense of trust. Either was SoMe may prompt the formulation of unbalanced judgments. Correctly managing SoMe communication is therefore crucial for physician. The rapid, fragmented, and often emotionally unfiltered nature of online communication places the clinician in a position where disorganized and opinionated information must be contended with. Such content must therefore be regulated and mediated by clear and effective rules [17].

### Self-promotion by professionals through social media

Self-promotion is often used for acquiring reputational capital and generating value. This process is largely developed through the strategic management of social relations that provide visibility, commitment, and competition. To evaluate the suitability of the web for self- promotion, health professionals need clarity on the following: the objectives and purposes of communication (such as studies, awareness raising, research interests, and clinical activities), the nature of the target audience (inreach vs. outreach), and the characteristics of the information shared (e.g., disclosure of preliminary results or nonpublic information).

Self-promotion, or personal branding, appears to be directed towards acquiring reputational capital and generating value. This process is largely developed through the strategic management of social relations that provide visibility, commitment, and competition [19]. Technological advances have led to greater ease of communication on the web, including through SoMe. This has resulted in a situation wherein careers are a personal brand that is also managed in the virtual arena, within a complex system of identity negotiation. SoMe is often used as a showcase, and this environment is rarely perceived as intended for developing communicative and relational practices with citizens and colleagues.

#### Professional identity

Self-promotion affects both the perception of oneself and the perception of the profession. The circular and transformative movement between platforms and users presupposes a relational dimension with a potential “public” of reference. Healthcare workers should therefore maintain professionalism on such platforms in a manner no different from that adopted during clinical practice [7].

#### Public engagement

Through self-promotion, physicians can become communicators, extending their capacity to convey scientific content beyond their peers to a broadened general public via the web. When doing so, the physician should respect the traditional principles that pertain to f the divulging of scientific and medical content.

### Appropriate use of social media for scientific updating and promotion

#### Statements

- 6.1 Scientific dissemination via SoMe should adhere to the same methodological, bioethical, and authoritative standards that govern the publishing of scientific research and practices. In the absence of these standards, content disseminated through SoMe should not be regarded as scientific.
  $$\left[Median\;score:\;8.5\;(IQR\;7-9);\;agreement:\;80\%\right]$$
- 6.2 As is the case with scientific research communicated through bibliometric methods, any content intended to argue, suggest, or demonstrate evidence must be supported by an appropriate bibliographic references. In the absence of this standard, content disseminated through SoMe should be considered the personal opinion of the author.
  $$\left[Median\;score:\;8\;(IQR\;7-9);\;agreement:\;80\%\right]$$
- 6.3 Dissemination of clinical data, whether personal, procedural, or administrative, in graphic or numerical form and even in aggregate form, requires a priori authorization from the patient. This authorization must accompany the disseminated information.
  $$\left[Median\;score\;:\;9\;(IQR\;7-9);\;agreement:\;100\%\right]$$

#### Rationale

The methodological aspects of scientific research and its dissemination are subjected to clear regulations. These regulations pertain even to contexts that could be considered “unconventional,” including the use of SoMe. Information disseminated through SoMe must adhere to the same standards of appropriateness, adequacy, safety, and verifiability as scientific content.

## Conclusions

Dissemination of medical and scientific information or data through social media must adhere to the same ethical, deontological, and scientific standards and principles as the traditional forms of dissemination. Clinicians must not disseminate practices, procedures, or data that are not supported by scientific evidence. Healthcare professionals must act responsibly and professionally when using social media for disseminating medical and scientific information. National and local authorities should determine regulations, laws, and minimal standards for the use of SoMe for disseminating medical and scientific information.

## Data Availability

No datasets were generated or analysed during the current study.

## Abbreviations

IQR: Interquartile range
SoME: Social media
